# Facile Synthesis of Silver Nanoparticles and Preparation of Conductive Ink

**DOI:** 10.3390/nano12010171

**Published:** 2022-01-05

**Authors:** Gui Bing Hong, Yi Hua Luo, Kai Jen Chuang, Hsiu Yueh Cheng, Kai Chau Chang, Chih Ming Ma

**Affiliations:** 1 Department of Chemical Engineering and Biotechnology, National Taipei University of Technology, Taipei 10608, Taiwan; lukehong@ntut.edu.tw (G.B.H.); d8906002@mail.ntust.edu.tw (Y.H.L.); 2 School of Public Health, College of Public Health and Nutrition, Taipei Medical University, Taipei 11490, Taiwan; kjc@tmu.edu.tw; 3 Department of Public Health, School of Medicine, College of Medicine, Taipei Medical University, Taipei 11490, Taiwan; 4 Department of Nursing, St. Mary’s Junior College of Medicine, Nursing and Management, Yi-Lan 26647, Taiwan; noidea@smc.edu.tw (H.Y.C.); amy03124@smc.edu.tw (K.C.C.); 5 Department of Cosmetic Application and Management, St. Mary’s Junior College of Medicine, Nursing and Management, Yi-Lan 26647, Taiwan

**Keywords:** silver nanoparticles, conductive ink, chemical reduction, electrical resistivity

## Abstract

In the scientific industry, sustainable nanotechnology has attracted great attention and has been successful in facilitating solutions to challenges presented in various fields. For the present work, silver nanoparticles (AgNPs) were prepared using a chemical reduction synthesis method. Then, a low-temperature sintering process was deployed to obtain an Ag-conductive ink preparation which could be applied to a flexible substrate. The size and shape of the AgNPs were characterized by ultraviolet–visible spectrophotometry (UV-Vis) and transmission electron microscopy (TEM). The experiments indicated that the size and agglomeration of the AgNPs could be well controlled by varying the reaction time, reaction temperature, and pH value. The rate of nanoparticle generation was the highest when the reaction temperature was 100 °C within the 40 min reaction time, achieving the most satisfactorily dispersed nanoparticles and nanoballs with an average size of 60.25 nm at a pH value of 8. Moreover, the electrical resistivity of the obtained Ag-conductive ink is controllable, under the optimal sintering temperature and time (85 °C for 5 min), leading to an optimal electrical resistivity of 9.9 × 10^−6^ Ω cm. The results obtained in this study, considering AgNPs and Ag-conductive ink, may also be extended to other metals in future research.

## 1. Introduction

Nanotechnology covers a wide range of fields (e.g., electronics, cosmetics, catalysis, industrial medicine, and sensor systems), and is currently one of the most popular disciplines in the field of scientific research [1,2,3,4]. Due to quantum effects, nano-grade matter differs from bulk-sized substances, in terms of physical and chemical properties, and has numerous novel applications [5,6]. Consequently, nanotechnology has received a significant amount of attention. In particular, nanotechnology has been employed for electronic semiconductors, medical biotechnology, environmental engineering, and energy, while electronic circuitry is continually being reduced to nanometer scale [7,8,9,10]. The application of nanoconductive metals in electronic circuitry has continued to attract attention. In addition to their high conductivity, the ductility of conductive metallic nanomaterials renders them suitable for flexible electronic products, such as smart watches and smart bracelets [11].

In the literature, a variety of physical and chemical synthetic methods have been reported for the preparation of nanoparticles, such as mechanical milling, chemical vapor deposition, mechanochemical synthesis, photochemical reduction, ion sputtering, hydrothermal method, laser ablation, and chemical reduction [3,12,13]. Chemical reduction methods are bottom-up synthesis methods, being some of the most commonly used methods for preparing metal nanoparticles. These are facile synthesis methods with easily controllable nanoparticle characteristics (e.g., function, size, and shape) [14]. However, the precursors, reducing agents (e.g., sodium citrate, Na_3_C_6_H_5_O_7_; ascorbic acid, C_6_H_8_O_6_; and sodium borohydride, NaBH_4_), stabilizers (e.g., polyvinylpyrrolidone, PVP; polyvinyl alcohol, PVA; and hexadecyl trimethyl ammonium bromide, CTAB), the reducing agent ratio, the stabilizer ratio, reaction time, reaction temperature, stirring rate, and pH value lead to major effects and pose various constraints in chemical synthetic methods [1,3]. Additionally, some reducing agents and stabilizers are highly toxic and, as such, may cause environmental pollution or risks in the production process. Studies on nanoparticles synthesized by chemical reduction have indicated that when weaker reducing agents—such as ascorbic acid, glucose, or sodium hypophosphite—are used for the chemical reduction, polyols are typically used as solvents to facilitate the reduction process. Meanwhile, when stronger reducing agents—such as sodium borohydride or hydrazine—are used, water is the most common solvent. Furthermore, composite stabilizers are typically used to synthesize smaller metal nanoparticles [1,15,16].

Conductive metal ink contains highly conductive micro- or nano-grade solid metal particles, which can cover or be printed onto conductive substrates [17,18]. A heating or chemical process is then used to increase the bonding density of the conductive materials, and the produced circuits have the ability to conduct electrical currents and adhere to plastic, glass, or paper substrates [19,20]. The basic requirements for conductive ink are that they are conductive, adhesive, and printable. At present, conductive ink is used for the printing of electronic components such as circuits, membrane switches, and radio frequency identification tags, as well as other electronic tags [9,11,21]. Conductive metal ink is mainly composed of a conductive metal, polymers, solvents, and additives. Gold and silver are commonly used conductive metals and, to avoid affecting their conductivity, post-processing must be performed in vacuum or an inert gas environment [22]. In terms of conductivity, silver, copper, and gold are the most conductive. Compared with gold and copper nanoparticles, silver nanoparticles have a lower price than gold and are not as easily oxidized as copper [23,24]. Therefore, the development of silver-based conductive ink has become a major research focus. However, in many of the methods for producing silver nanoparticles, most of the chemicals used are toxic, which may lead to environmental pollution. Traditional conductive ink printing technologies, such as spin coating and blade coating, have complicated processes and lead to raw material being wasted. In addition, in the final sintering step of printed electronic products, it is necessary to remove the solvent and form a conductive path between the silver nanoparticles, in order to enable the smooth transfer of electrons. This usually requires extremely high temperatures and high energy consumption [25,26]. With the development of the electronic technology industry, flexible electronic products are now the primary goal of development; however, substrates such as polyethylene terephthalate (PET), paper, and textile materials are usually not resistant to high temperatures. Therefore, the development of non-toxic, environmentally friendly, and low-temperature sintering processes has become a current research trend.

In our past research [27], effects of the reducing agent and stabilizer on nanoparticle generation had been studied, and we had tested the properties of the ink and the effect of the sintering agent concentration on conductive patterns. In this study, silver nanoparticles were prepared by chemical reduction, and environmentally-friendly synthetic methods using chemical additives that are non-toxic and which have no negative impact on the environment were selected, in an attempt to prepare a product with a single morphology, size, crystal state, and good dispersion. The influence of multiple experimental variables during the preparation process, including reaction time, reaction temperature, rotation speed, and pH value, on the size, shape, and stability of the prepared nanoparticles is fully discussed. The synthesized silver nanoparticles were characterized by ultraviolet–visible spectrophotometry (UV-Vis), transmission electron microscopy (TEM), and X-ray diffraction (XRD). In addition, in order to prepare a conductive ink that can be stably dispersed for a long time and is advantageous for inkjet printing and low-temperature calcination, we add a spontaneous sintering agent and a commercial dispersant and print on a PET flexible substrate. Furthermore, we discuss the effects of the number of inkjets, sintering temperature, and sintering time on the final electrical conductivity.

## 2. Materials and Methods

### 2.1. Materials

Sodium chloride (purity ≥ 99.5%), sodium hydroxide (NaOH, purity ≥ 97%), silver nitrate (AgNO_3_, purity ≥ 99.8%), hydrochloric acid (36.5–38.0%), and nitric acid (68.0–70.0%) were used without further purification and purchased from Fisher Chemical (Pittsburgh, PA, USA). Propane-1,2-diol (purity ≥ 99.5%), L-Ascorbic acid (C_6_H_8_O_6_, purity ≥ 99.7%), and sodium citrate (Na_3_C_6_H_5_O_7_, purity ≥ 99%) were obtained from Acros Organics (Geel, Belgium). Commercial wetting agent (type 2223) and dispersant agent (type 1186) were used in this study, supplied by Marvel Chemical Co. (Taipei, Taiwan). All chemicals were analytical grade and used as received.

### 2.2. Preparing the AgNPs and Ag Conductive Ink

The equipment (e.g., beaker, volumetric pipet, thermometer, stir bar, test tubes, volumetric flask) was rinsed using deionized water, in order to eliminate residual chemical substances. Subsequently, 500 mL of 0.001 M AgNO_3_ was placed in a beaker, covered with aluminum foil, and the solution was stirred using a magnet at an appropriate rotation speed ranging from 200 to 600 rpm. The solution was then heated to boiling, and appropriate amounts of Na_3_C_6_H_5_O_7_ (reducing agent) and C_6_H_8_O_6_ (stabilizer) were immediately added, the sample obtained using the AgNO_3_:Na_3_C_6_H_5_O_7_ ratio 1:6 (i.e., no added stabilizer), the solution was stirred and heated continuously ranging from 10 to 40 min. The sample obtained using the AgNO_3_:Na_3_C_6_H_5_O_7_:C_6_H_8_O_6_ ratio 1:6:1.0 (i.e., added stabilizer), reaction temperature (20, 60, and 100 °C), and the pH of the solution was adjusted ranging from 3 to 9 by adding 0.2 M NaOH and nitric acid solution. The solution was then stirred continuously and cooled to room temperature, to complete preparation of the colloid solution of AgNPs.

In order to obtain a high-quality Ag conductive ink, the AgNP solution underwent centrifugation at high speed, which formed black sediments which were then washed several times with deionized water and collected through a glass funnel filter. The centrifugation speed is 13,000 rpm, 10 min each time. The nanoparticle sediment was dispersed into a mixed solution of deionized water and propane-1,2-diol. The volume ratio between propylene glycol and water was 41:59. The AgNP mixed solution was then added to a solution of commercial dispersant and wetting agent. In the end, Ag conductive inks were obtained after being mixed for 30 min using an ultrasonicator, thus completing the preparation of the conductive ink with a AgNP concentration of 30 wt.%. The approximated composition of the commercial wetting and dispersing agents include isotridecyl alcohol, ethoxylate, phosphate, tetrapropylene benzene, and were mixed at a ratio of 2:1. In addition, dispersing and wetting agents with an additive ratio of 0.7 wt.% were used in this study.

The AgNP solution was rinsed using deionized water and centrifuged twice at high speed. The nanoparticle sediment was dispersed into a mixed solution of deionized water and propane-1,2-diol. The solution was then added to a mixed solution of commercial dispersant and wetting agent, then evenly mixed for 30 min using an ultrasonicator (DC150, Delta, Taipei, Taiwan), thus completing the preparation of the Ag conductive ink. The approximated composition of the commercial wetting and dispersing agents include isotridecyl alcohol, ethoxylate, phosphate, and tetrapropylene benzene.

### 2.3. Printing Conductive Patterns

**Figure 1:**
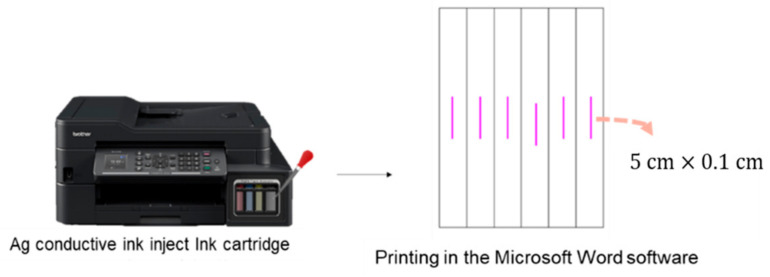
The schematic diagram of printing conductive pattern.

An ink cartridge for an MFC-T910DW printer (Brother International Corporation, Tokyo, Japan) was filled with the prepared Ag conductive ink. A printing conductive pattern test was designed using 5.0 × 0.1 cm^2^ rectangular samples for printing in the Microsoft Word software (Office 2016, Microsoft Corporation, Redmond, WA, USA), as shown in Figure 1. Pieces of inkjet PET paper were then placed in the printer, and repeat printing was carried out in accordance with the requirements of the experiment.

### 2.4. Characteristics of the Nanoparticles and Electrical Properties of Conductive Ink Material

In this study, the AgNP solution samples without any rinsing were directly analyzed by an UV-Vis (Gensys 10 Series, Thermo Scientific, Waltham, MA, USA). The morphological properties of the Ag nanoparticles were studied using an H-7100 (Hitachi, Tokyo, Japan) TEM. XRD (Empyrean, Malvern Panalytical, Malvern, UK) was used to further characterize the nanoparticles. Measurements of the Ag conductive ink viscosities were carried out using a DV3T viscometer (Brookfield Engineering Laboratories, Middleboro, MA, USA). The surface tension of Ag conductive ink was assessed using Model 100SB (Sindatek Instruments, New Taipei, Taiwan). Finally, electrical resistivity of the patterns was measured using a Surfcorder ET3000 microfigure measuring instrument (Kosaka Laboratory, Tokyo, Japan) and a Keithley 2000-EM4P four-point probe analyzer (Tektronix, Beaverton, OR, USA).

## 3. Results and Discussion

**Figure 2:**
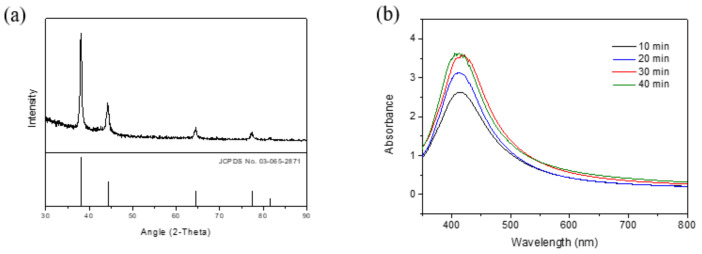
(**a**) The X-ray diffraction (XRD) spectra of the prepared nanoparticles; and (**b**) UV-Vis maps obtained for samples prepared using various reaction times.

The prepared nanoparticles were tested by XRD, and the results are shown in Figure 2a. The analysis results show five characteristic peaks, the plane coordinates of which are 38.11°, 44.30°, 64.44°, 77.39°, and 81.54°, respectively. Comparing them with JCPDS No. 03-065-2871, the analysis results indicate the crystalline structure of silver. In our past research, we have experimentally explored the effects of the reducing agent and stabilizer on nanoparticle generation [27]. When the stabilizer was added at the precursor:reducing agent:stabilizer molar ratio of 1:6:1, the Ag nanoparticles exhibited satisfactory dispersibility, and the stabilizer was confirmed to increase the thickness of the surface functional base layers of the nanoparticles, thus enabling their individual dispersion.

### 3.1. Synthesis of Ag Nanoparticles

#### 3.1.1. Effect of the Reaction Time

**Figure 3:**
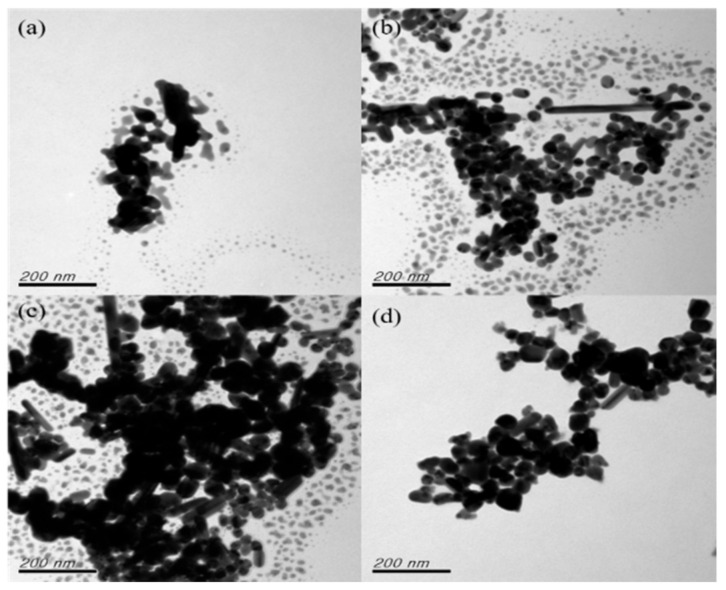
TEM images obtained for samples prepared using various reaction times: (**a**) 10 min; (**b**) 20 min; (**c**) 30 min; and (**d**) 40 min.

The reaction time is one of the most important factors influencing the growth and aggregation of silver nanoparticles. It can be controlled not only by selection of the reducing agent, but also by the reaction time of the chemical reduction process [28,29,30]. L-Ascorbic acid was employed as the stabilizer in this study. In our past research, the Ag nanoparticles exhibited satisfactory dispersibility, and the stabilizer was confirmed to increase the thickness of the surface functional base layers of the nanoparticles, thus enabling their individual dispersion. In order to avoid interference from stabilizers, the effect of the reaction time was studied without ascorbic acid. With the AgNO_3_:Na_3_C_6_H_5_O_7_ molar ratio fixed at 1:6, the effect of reaction time (10, 20, 30, or 40 min) on nanoparticle generation was examined. The reaction temperature and rotation speed were fixed at 100 °C and 300 rpm, respectively. As illustrated in Figure 2b, an increase in the reaction time led to a rise in the peak intensity, indicating that more nanoparticles were generated. A very slight impact effect on peak position was observed from 10 to 40 min, revealing little effect on particle size. However, a slight peak shift at 30 min reaction time could be observed and no considerable difference in peak intensity from 30 min to 40 min reaction time was found. Accordingly, the optimal reaction time was determined to be 40 min. Figure 3 presents the TEM results for the nanoparticle samples and reaction times ranging from 10 to 40 min. Figure 3a–c show that the nanonuclei were not thoroughly developed. Figure 3a–c also shows that the nanonuclei grow and become slight larger over time. At the 40 min reaction time (Figure 3d), no nanonuclei were observed, indicating that all the nanonuclei had developed into comprehensive nanoparticles, and the agglomeration of nanoparticles was also observed.

#### 3.1.2. Effect of Reaction Temperature

**Figure 4:**
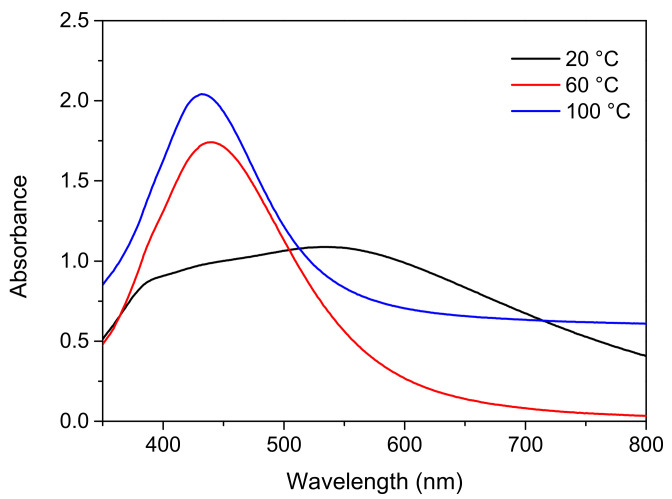
UV-Vis analysis results obtained for samples prepared at various reaction temperatures.

Besides affecting the yield of nanoparticles, the reaction temperature plays a crucial role in determining the nanoparticle size [31,32]. With the AgNO_3_:Na_3_C_6_H_5_O_7_:C_6_H_8_O_6_ ratio fixed at 1:6:1, the effect of reaction temperature (20, 60, and 100 °C) on the growth of the nanoparticles was examined. The rotation speed and reaction time were fixed at 300 rpm and 40 min, respectively. Figure 4 illustrates the UV-Vis analysis results. When the reaction temperature was 20 °C, the waveforms showed no characteristic absorption peaks from the Ag nanoparticles. Accordingly, after 40 min of reaction, no Ag nanoparticles were generated. When the reaction temperature was 60 or 100 °C, characteristic absorption peaks were identified at 432 and 438 nm, respectively, indicating the generation of Ag nanoparticles. Moreover, blue shifts were identified at higher reaction temperatures, revealing that the size of the nanoparticles decreased when the temperature was increased. The peak intensity was highest for 100 °C, indicating that the most nanoparticles were generated at this temperature.

**Figure 5:**
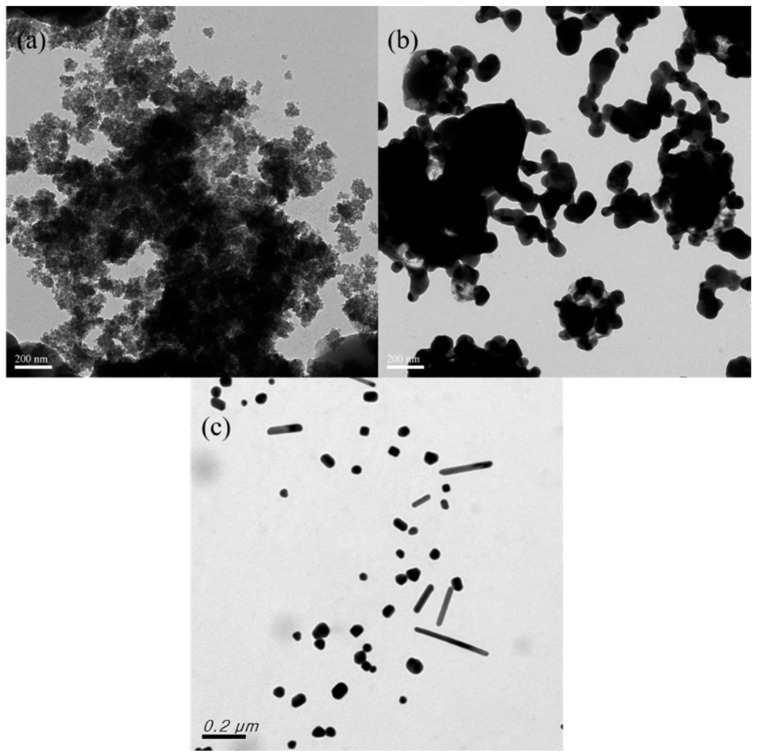
TEM images obtained for samples prepared at various reaction temperatures: (**a**) 20 °C; (**b**) 60 °C; and (**c**) 100 °C.

Figure 5 depicts the TEM analysis results for the samples prepared at different reaction temperatures. No noticeable nanoparticles were generated at 20 °C, but many underdeveloped nanonucleus agglomerates were identified (Figure 5a). Accordingly, after 40 min of reaction, the nanoparticles remained in the nucleation stage. When the reaction temperature was 60 °C (Figure 5b), nanoparticle agglomeration was severe, and many clots were identified. Conversely, when the reaction temperature was 100 °C (Figure 5c), the nanoparticles were fully developed and satisfactorily dispersed, and their forms were primarily nanoballs and nanopillars. According to the UV-Vis and TEM results, at reaction temperatures of 60 and 100 °C, noticeable characteristic absorption peaks could be identified, indicating the generation of Ag nanoparticles; however, the nanoparticles generated at 60 °C exhibited poor dispersibility. Therefore, the optimal reaction temperature was determined to be 100 °C. According to the TEM results, the reaction temperature significantly affected the rate of nanoparticle generation as well as the morphology and dispersibility of the nanoparticles.

#### 3.1.3. Effect of Rotation Speed

**Figure 6:**
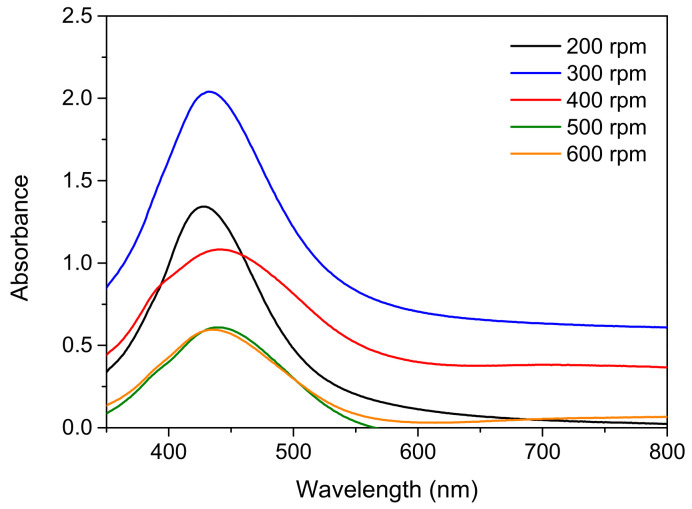
UV-Vis analysis results obtained for samples prepared at various rotation speeds.

Rotation speed also affects the nanoparticle generation in silver reduction methods [33]. With the AgNO_3_:Na_3_C_6_H_5_O_7_:C_6_H_8_O_6_ ratio fixed at 1:6:1 and the reaction temperature and time fixed at 100 °C and 40 min, respectively, the effect of rotation speed on nanoparticle generation was examined. Figure 6 displays UV-Vis maps obtained at various rotation speeds. The peak intensity was considerably higher at a rotation speed of 300 rpm than at 200 rpm, indicating that more nanoparticles were generated. The slight red shift in the peak position also suggested slightly larger nanoparticles. When the rotation speed was 400 rpm, the peak intensity was considerably lower than for 300 rpm, signifying substantially less nanoparticle generation.

**Figure 7:**
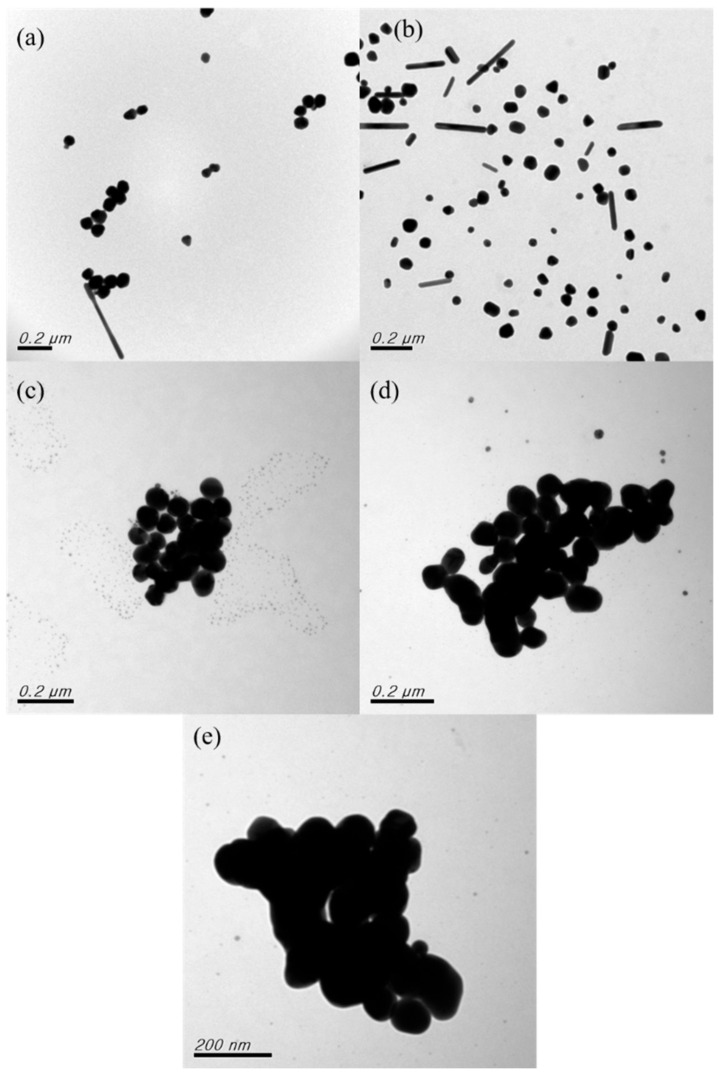
TEM images obtained for samples prepared at various rotation speeds: (**a**) 200 rpm; (**b**) 300 rpm; (**c**) 400 rpm; (**d**) 500 rpm; and (**e**) 600 rpm.

Figure 7 presents the TEM analysis results. As shown in Figure 7a,b, with rotation speeds of 200 and 300 rpm, the nanoparticles exhibited satisfactory dispersibility and no agglomeration was observed. The primary forms of the nanoparticles were nanoballs and nanopillars. As illustrated in Figure 7c, when the rotation speed was increased to 400 rpm, nanoparticle agglomeration occurred and the particles were considerably larger. Underdeveloped nanonuclei also formed. When the rotation speed was 500 rpm, the agglomeration was more severe (Figure 7d). Finally, when the rotation speed was 600 rpm (Figure 7e), clots formed among the Ag nanoparticles. In summary, the TEM analysis results revealed that the nanoparticles prepared at the rotation speeds of 200 and 300 rpm exhibited satisfactory dispersibility and no agglomeration, while the UV-Vis analysis revealed that the number of nanoparticles generated was the highest at 300 rpm. Therefore, 300 rpm was determined to be the optimal rotation speed, and the rotation speed was confirmed to strongly affect nanoparticle generation.

#### 3.1.4. Effect of pH

**Figure 8:**
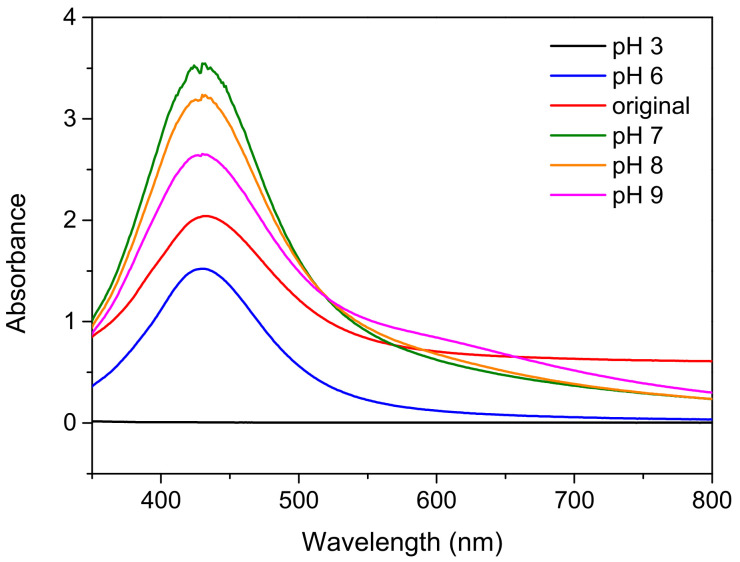
UV-Vis analysis results obtained for samples prepared at various pH values.

Previous research [6,26] has clearly demonstrated that the electrical and optical performance of AgNPs is influenced by particle size, which is, in turn, influenced by the pH. With the AgNO_3_:Na_3_C_6_H_5_O_7_:C_6_H_8_O_6_ ratio fixed at 1:6:1 and the reaction time, reaction temperature, and rotation speed fixed at 40 min, 100 °C, and 300 rpm, respectively, 0.2 M of NaOH and HNO_3_ was applied to adjust the pH of the Ag nanoparticle solution, and the effect of pH (pH 3, 6, 7, 8, or 9) on nanoparticle generation was investigated. Figure 8 presents UV-Vis maps obtained for samples prepared using various pH values. When the pH was adjusted to 6, the peak intensity was lower than that of the original solution without adding NaOH or HNO_3_, but the positions of the characteristic peaks were not considerably different. When the pH was adjusted to 3, no substances were detected in the UV-Vis map, and the solution was clear. This indicated that no nanoparticles were generated under strong acidic conditions. When the pH was adjusted to 7, the peak intensity was considerably higher than for a pH of 6. When the pH was adjusted further to 8 or 9, the peak intensity was lower than for a pH of 7, indicating lower nanoparticle generation; however, the characteristic peak positions were unchanged, revealing no difference in particle size.

**Figure 9:**
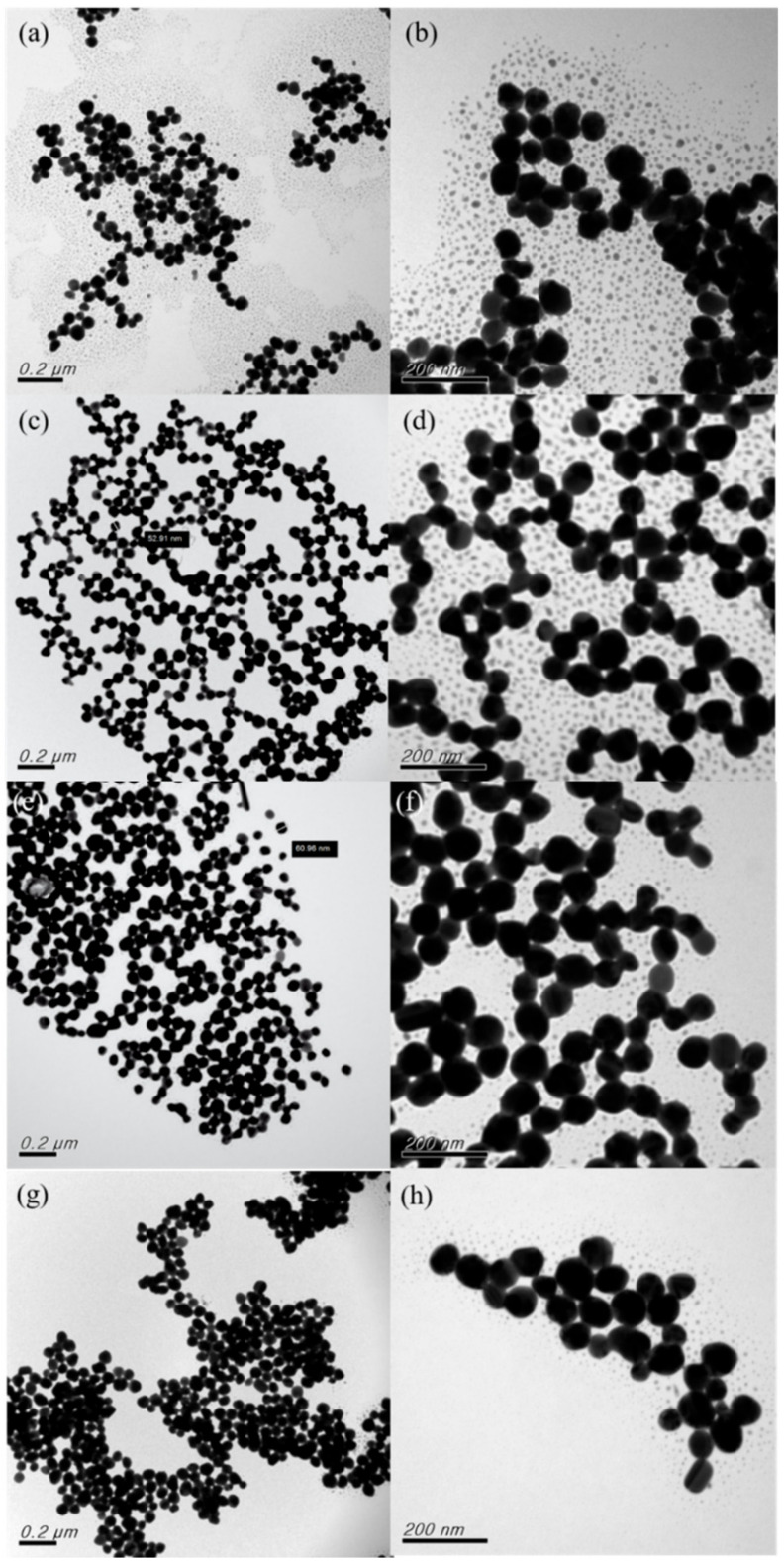
TEM images at two magnifications obtained for samples prepared with pH of: (**a**,**b**) 6; (**c**,**d**) 7; (**e**,**f**) 8; and (**g**,**h**) 9.

Figure 9 presents the TEM analysis results for various pH values. When the pH was adjusted to 6, numerous large under-developed nanonuclei were observed alongside fully developed nanoparticles (Figure 9a,b). When the pH was adjusted to 7, no nanonuclei were observed in the low-magnification TEM images (Figure 9c); furthermore, the dispersibility was satisfactory, and the average particle size was 54.93 nm. However, the high-magnification TEM images showed that the nanonuclei remained around the nanoparticles (Figure 9d). When the pH was adjusted to 8, the nanoparticles exhibited favorable dispersibility, and their average size was 60.25 nm (Figure 9e). High-magnification TEM (Figure 9f) revealed that nearly no residual nanonuclei existed. This may have been because the nanoparticles were thoroughly developed through Ostwald ripening when the pH was adjusted to 8. When the pH was 9, nanoparticle agglomeration was observed. Moreover, after pH adjustment, all the nanoparticles were nanoballs, and no nanopillars coexisted with the nanoballs [33]. This indicates that pH influenced the forms of the nanoparticles considerably [34]. In summary, the UV-Vis analysis results revealed that the peak intensity was highest for pH 7, followed by pH 8; as such, the number of nanoparticles generated was highest at these pH values. According to the TEM results, the nanonuclei were underdeveloped for pH 7 and fully developed into nanoparticles only when the pH was 8. Thus, a pH of 8 was determined as the optimal pH.

### 3.2. Ag Conductive Ink

#### 3.2.1. Effect of Sintering Temperature and Time

The basic requirements for conductive ink are that they are conductive, adhesive, and printable. The low-toxicity and environmentally-friendly conductive inks approach has become a popular trend. As indicated by the literatures [10,19,20,21], ethylene glycol, propylene glycol, isopropanol, and glycerin are commonly used as conductive ink mixed solvents. Because this study uses a printer for inkjet printing, a lower viscosity ink characteristics are required. Hence, propylene glycol and deionized water were selected as the solvents for the conductive ink. In our past research, the amount of Ag nanoparticles added to the ink was fixed at 30 wt%, and a volume ratio of 41:59 between propylene glycol and water was determined to be the optimal solvent ratio [27]. The viscosity and surface tension of the conductive ink are 3.19 mPa s and 0.71 mN m^−1^, respectively.

**Figure 10:**
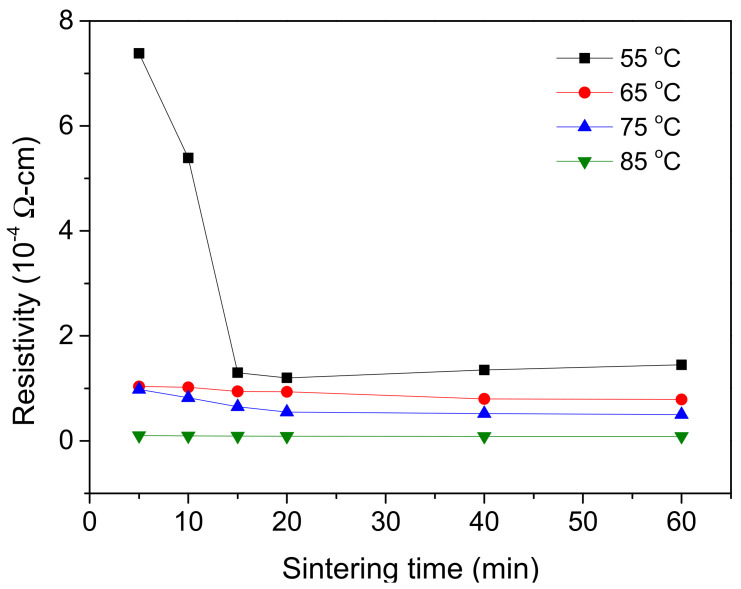
Effect of sintering temperature and time on electrical resistivity.

The sintering agent (NaCl) could improve conductivity after sintering at low temperature. With the NaCl concentration fixed at 70 mM and the number of times of repeated printing fixed at five, the effects of sintering temperature (55, 65, 75, and 85 °C) and time (5, 10, 15, 20, 40, and 60 min) on the electrical resistivity of the sample patterns were determined. After the tests, the samples were cooled to room temperature and a four-point probe was used to test their electrical resistivity (Figure 10). The electrical resistivity of the samples was found to be substantially lower after they had been sintered at 55 °C for 15 min. This was because water evaporation caused an increase in the NaCl concentration, leading to the nanoparticles forming a conductive medium after being sintered. The electrical resistivity was thus decreased, enabling smooth electron transmission. The changes in electrical resistivity were relatively small when sintering was performed for only 15 min. When the samples were sintered at 65 °C for 5 min, their electrical resistivity was 1.037 × 10^−5^ Ω cm—seven times lower than that of the samples sintered at 55 °C for 5 min. When the sintering time was 20 min, the decrease in electrical resistivity became relatively small: the resistivity was 9.36 × 10^−5^ Ω cm. The decrease in resistivity only became noticeable when the sintering time was increased to 40 or 60 min. When the sintering temperature was 75 °C, the electrical resistivity did not differ substantially from that when the sintering temperature was 65 °C, but did decrease as the sintering time increased. When the sintering temperature was 85 °C, the electrical resistivity was at its lowest for all four temperature-related samples when sintering was performed for 5 min (9.9 × 10^−6^ Ω cm), and the electrical resistivity continued to decrease as the sintering time increased.

In consideration of the experimental results and practical factory production conditions, the use of energy to raise sintering temperature must be minimized. Therefore, 65 °C and 20 min were determined to be the optimal sintering temperature and time, respectively. The resulting electrical resistivity value was close to the values reported by Cao et al. (2017) [26] (5.01 × 10^−3^ Ω cm) and Ahn et al. (2011) [28] (1.22 × 10^−4^ Ω cm). Additionally, the advantages of the conductive ink presented in this work include low-temperature sintering and stable dispersion for a long time.

#### 3.2.2. Effect of Printing Times

**Figure 11:**
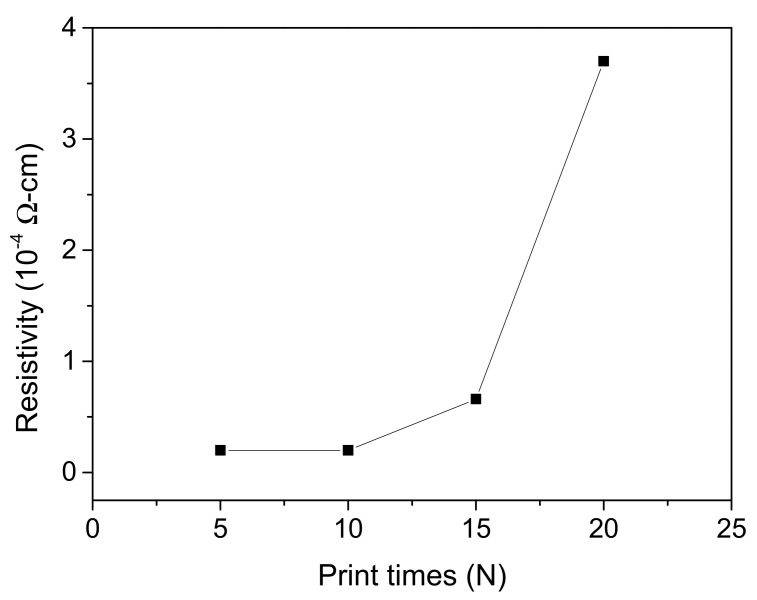
Effect of printing times on electrical resistivity.

Printing time is one of the most important factors influencing the resistivity and the thickness of the printed conductive patterns [35]. In this work, the printed conductive patterns were prepared by printing 5, 10, 15, and 20 times, and the experiments were sintered at a temperature of 65 °C for 20 min. The relationship between the electrical resistivity value and the number of printing cycles was studied. As shown in Figure 11, the electrical resistivity of the printing conductive patterns increased with the number of printing cycles. The electrical resistivity of the conductive patterns after 20 printing cycles rose sharply, to approximately 3.7 × 10^−4^ Ω cm. This may be interpreted as indicating that, when the number of printing cycles is increased, the amount of solvents and additives on the conductive pattern is also increased, and the remaining solution on the conductive pattern is not completely dried. Thus, the electrical resistivity of the conductive patterns increases sharply along with the number of printing cycles.

## 4. Conclusions

When the precursor and reducing agent were added in the molar ratio 1:6 for a 40 min reaction, the number of fully developed Ag nanoparticles of various shapes was maximized, and the particle size was minimized. The rate of nanoparticle generation was highest when the reaction temperature was 100 °C, enabling the full development of nanoparticles within the 40 min reaction time. Rotation speed was confirmed to strongly influence the generation and agglomeration of nanoparticles, and 300 rpm was determined to be the optimal rotation speed. The optimal pH value was determined to be 8, as this resulted in the most fully developed nanoparticles, the most satisfactorily dispersed nanoparticles, and nanoballs with an average size of 60.25 nm.

Moreover, the electrical resistivity decreased as the sintering temperature and time increased; when the samples were sintered at 85 °C for 5 min, their electrical resistivity was 9.9 × 10^−6^ Ω cm. The proposed conductive ink, created using new ingredients and manufacturing methods, can be used to fabricate ultrafine circuits or special circuits to replace antennae. Such specially processed conductive ink can be used to print circuits. The application of conductive ink also improves resource efficiency in manufacturing. The excessive use of water resources and subsequent wastewater treatment can be reduced, leading to enhanced manufacturing process simplicity, cost reduction, and efficiency improvement.

## Data Availability

Not applicable.
